# Miso Soup Consumption Enhances the Bioavailability of the Reduced Form of Supplemental Coenzyme Q_10_

**DOI:** 10.1155/2020/5349086

**Published:** 2020-01-07

**Authors:** Michiyo Takahashi, Mayumi Nagata, Takehiko Kaneko, Toshikazu Suzuki

**Affiliations:** ^1^Graduate School of Human Ecology, Wayo Women's University, 2-3-1 Konodai, Ichikawa, Chiba 272-8533, Japan; ^2^Department of Health and Nutrition, Wayo Women's University, 2-3-1 Konodai, Ichikawa, Chiba 272-8533, Japan

## Abstract

Coenzyme Q_10_ (CoQ_10_) is an essential compound that is involved in energy production and is a lipid-soluble antioxidant. Although it has been proposed as an antiaging and a health-supporting supplement, its low bioavailability remains a significant issue. Concurrent food intake enhances the absorption of orally administered CoQ_10_, but it has not been fully established whether specific food substances affect intestinal CoQ_10_ absorption. Therefore, to determine whether the bioavailability of supplemental CoQ_10_ is affected by diet, P30, a granulated and reduced form of CoQ_10_, was dispersed in four different foods, clear soup, miso soup, milk soup, and raw egg sauce. Those foods which contained CoQ_10_ were consumed on different occasions at intervals of 6–14 weeks by the same participants. Thirteen participants were recruited in the single-dose and repeated clinical study. When miso soup containing P30 was provided, the serum CoQ_10_ concentration increased faster than when participants consumed other P30-containing soups or a P30-containing raw egg sauce. The area under the curve for serum CoQ_10_ during the first 5 h after consumption of the P30-containing miso soup was approximately 1.5 times larger than those after the consumption of other P30-containing meals. These data imply that the absorption of CoQ_10_ supplements can be enhanced by consuming them with food and in particular with specific food substances, such as miso soup.

## 1. Introduction

Coenzyme Q_10_ (CoQ_10_), a vitamin-like substance, is involved in energy production and is a lipid-soluble antioxidant [1–3]. Many studies have reported a relationship between CoQ_10_ and aging. For example, the amount of CoQ_10_ in muscles and organs decreases with age [4], as does the serum CoQ_10_ concentration and that of the reduced form [5] in healthy adults. Both blood levels and the ratio of the reduced form of CoQ_10_ concentration to total coenzyme Q_10_ in hospitalized elderly people were lower than that in the healthy elderly people [6]. Also, many studies have shown associations between serum CoQ_10_ status and health [7–11]. In addition, CoQ_10_ supplementation has been shown to ameliorate the symptoms of some geriatric disorders and to improve the quality of life of humans and some laboratory animals. CoQ_10_ supplementation ameliorates high blood pressure [12], glucose metabolism in diabetes [13], and the symptoms of Parkinson's disease [14] and reduces peripheral oxidative stress and inflammation in interferon *β*-1a-treated multiple sclerosis [15]. It can also increase the vitality of patients undergoing medical treatment and of the elderly residents of nursing homes [16–18]. Furthermore, it alleviates fatigue in patients with chronic fatigue syndrome [19, 20], hyperlipidemia [21], and in those with end-stage heart failure awaiting cardiac transplantation [22]. In older rats, CoQ_10_ supplementation has been shown to alleviate diabetes-induced learning and memory deficits and to improve cognitive performance when administered at a high dose [23]. In the senescence-accelerated prone 8 mouse, CoQ_10_ supplementation counteracts the deleterious effects of physical exercise-derived reactive oxygen species, improving mitochondrial function [24]. Thus, CoQ_10_ may be useful as an antiaging and health-supporting supplement.

In general, the absorption of compounds from the gastrointestinal tract is one of the most important determinants of oral bioavailability. Intestinal absorption of supplemental CoQ_10_ is slow and limited because of the compound's hydrophobicity and high molecular weight. Many types of CoQ_10_ delivery system have been developed that aim to increase the bioavailability of supplemental CoQ_10_, such as self-emulsifying drug delivery systems, nanotechnology-based drug delivery systems, cyclodextrin complexes, CoQ_10_-solanesyl poly(ethylene glycol) succinate micelles, and a reduced form of CoQ_10_ that is both emulsified and solubilized [25–27]. In addition, the concurrent consumption of food enhances the rate of the absorption of orally administered supplemental CoQ_10_ [28]. Therefore, the consumption of CoQ_10_-fortified foods may be a useful way of increasing the bioavailability of CoQ_10_.

It is well known that the absorption of nutrients, such as vitamins and minerals, is affected by the food items concurrently consumed or their components. For example, the enhancement of *β*-carotene absorption by mayonnaise consumption [29], of carotenoid absorption by avocado or avocado oil consumption [30], of vitamin E absorption by egg consumption [31], and of nonheme iron absorption by meat protein and vitamin C consumption, have been reported [32–34]. Conversely, some dietary fibers suppress the absorption of *β*-carotene, lycopene, and lutein [35]. Additionally, tannins, phytic acid, polyphenols, and calcium inhibit the absorption of nonheme iron [36–39]. Thus, the absorption of supplemental CoQ_10_ may also be affected by the food or a component with which it is consumed, although no previous studies have addressed this issue. Therefore, knowledge of the food items that could enhance the absorption of orally administered CoQ_10_ is crucial for the development of appropriate functional CoQ_10_-fortified foods.

Previously, we investigated the association between dietary habits and serum CoQ_10_ levels before and after long-term supplementation with a reduced form of CoQ_10_ [40, 41]. People with higher basal serum CoQ_10_ concentrations tended to consume more soy products [40], and those who had a higher increase in serum CoQ_10_ concentrations after the 1-year supplementation tended to consume more dairy products and eggs [41]. These results remind us that soy products, dairy products, and eggs might positively affect the absorption of CoQ_10_ supplements.

In this study, we investigated the effect of various foods on the bioavailability of supplemental CoQ_10_ using P30, a granulated and reduced form of CoQ_10_ supplement, and a typical Japanese meal, consisting of steamed rice, grilled salmon with marinated Japanese radish, boiled spinach, and soup. P30 was suspended in the soup or raw egg sauce in advance, before being provided to the participants. Miso soup, milk soup, and raw egg sauce were used as foods containing soy products, daily products, and eggs, respectively. Clear soup, which is seasoned with salt, was used as a reference food. The soup consumed was different in each experiment. Serum CoQ_10_ concentration was determined before, and 1.5, 3, and 5 h after eating the meals, and the bioavailability of CoQ_10_ was compared among the various foods that were concurrently consumed.

## 2. Materials and Methods

### 2.1. CoQ_10_ Supplements

A granulated, solubilized, and reduced form of CoQ_10_ supplement, P30, was used in the study. P30 contains 30 w/w% of reduced CoQ_10_ (120 mg per sachet), dextrin, gum Arabic, and L-ascorbate. These supplements were provided by the Kaneka Corporation (Osaka, Japan).

### 2.2. Study Design

Thirteen healthy volunteers (1 man and 12 women), who were students or staff at Wayo Women's University, participated in the study. Each participant took a CoQ_10_ supplement with a meal on four occasions, with the food type containing the CoQ_10_ differing on each occasion.  Table 1 shows the meal composition, the sauce of the CoQ_10_, the nutrient content of the meals. Using the data by Kubo et al. [42], CoQ_10_ content obtained from the meals in the clear soup, miso soup, milk soup, and raw egg source experiments were estimated as 0.52, 0.55, 0.56, and 0.59 mg, respectively, and these were the less than one two-hundredth of the CoQ_10_ supplements ingested. It suggests that the effect of CoQ_10_ content in meals itself was vanishingly low. The number of participants in each experiment is shown in [Supplementary-material supplementary-material-1]. Photos of the meal components provided for the subjects are shown in [Supplementary-material supplementary-material-1]. In the experiments, each participant ingested 120 mg of reduced CoQ_10_ (a sachet of P30) suspended in the food (indicated by red arrowheads in [Supplementary-material supplementary-material-1]). In the meals containing P30, the ratio of the concentrations of the reduced form of CoQ_10_ concentration to total coenzyme Q_10_ was >99%. To minimize the number of confounding factors, the same meal was consumed in each experiment, with the exception of the food item containing the P30.

The nutrient content of the meals was estimated using Calorie Make software (Toyo System Science Co., Ltd., Yokohama, Japan). The consumption of the meals started around 12:00 h, at least 3 h after breakfast. The control and test experiments were performed between March and December 2018 at intervals of 6–14 weeks. All subjects gave their informed consent for inclusion before they participated in this study. This study was conducted in accordance with the Declaration of Helsinki, and the protocol was approved by the Wayo Women's University Human Research Ethics Committee (no. 1734).

### 2.3. Blood Collection and CoQ_10_ Measurements

Blood samples were drawn from a vein at the baseline (just before meal ingestion), and 1.5, 3, and 5 h after the start of the meal, and serum was obtained by centrifugation after clot formation. Quantitative analysis of serum CoQ_10_ concentration was measured by Kaneka Techno Research Co., Ltd., using liquid chromatography with tandem mass spectrometry (LC/MS/MS) [43, 44]. In brief, 0.7 mL of the isopropanol was added to 0.1 mL of serum, mixed, and stored at −80°C until just before the analysis. After centrifugation, the supernatant was filtered through a membrane filter. Then, 200 *μ*L aliquots were mixed with 200 *μ*L of methanol and 50 *µ*L of oxidized CoQ_9_ (50 ng/mL in 2-propanol) as an internal standard and used as the sample for LC/MS/MS, which was performed using an AB Sciex Triple Quad 5500 LC-MS/MS system and a reversed-phase octadecyl-silica column (AB Sciex, Framingham, MA, USA). The intra- and interday coefficients of variation for CoQ_10_ were less than 2 and 10%, respectively.

### 2.4. Data Analysis

The increase in concentration of CoQ_10_ in the serum after the ingestion of a CoQ_10_-suspended test meal (ΔCoQ_10_) was calculated by subtracting the baseline value, and numerical data are expressed as mean ± SD. To compare the bioavailabilities of CoQ_10_, the areas under the serum CoQ_10_ concentration-time curves up to 5 h after ingestion (ΔAUC_0–5_) were calculated. These data were analyzed using one-way analysis of variance (ANOVA) with unpaired and repeated measures, and the differences between the means were evaluated by Holm–Bonferroni post hoc testing using js-STAR ver. 9. 3. 0j web application software (http://www.kisnet.or.jp/nappa/software/star/). *P* < 0.05 was considered to represent statistical significance.

## 3. Results

Thirteen healthy volunteers (1 man and 12 women) participated in the study. Each ingested a CoQ_10_ supplement in the form of suspended a suspension in a food item on up to four separate occasions. Twelve participated in the first experiment (supplemental CoQ_10_ in clear soup as a reference), thirteen participated in the second experiment (supplemental CoQ_10_ in miso soup), nine participated in the third experiment (supplemental CoQ_10_ in milk soup), and thirteen participated in the fourth experiment (supplemental CoQ_10_ in a raw egg sauce). The participation of each individual and the increases in serum total CoQ_10_ concentration after each meal are shown in Tables [Supplementary-material supplementary-material-1] and [Supplementary-material supplementary-material-1], respectively. ΔAUC_0–5_ was also calculated for each participant ([Supplementary-material supplementary-material-1]).

First, the increase in serum total CoQ_10_ (ΔCoQ_10_) concentration after each of the four experiments were compared using unpaired one-way ANOVA and Holm–Bonferroni post hoc testing because some participants missed in the first and third experiments. The concentration achieved following the consumption of P30 in miso soup was significantly higher than that achieved following consumption of the raw egg sauce after 1.5 h and higher than that achieved following all three other meals after 3 h ([Supplementary-material supplementary-material-1]). There were significant differences in ΔAUC_0–5_ between the clear soup and miso soup days, and between the miso soup and raw egg sauce days ([Supplementary-material supplementary-material-1]). The ΔAUC_0–5_ following miso soup ingestion was 4.44 ± 1.40 *μ*mol h/L, which is approximately 1.5- and 1.6-fold higher (*P* < 0.05) than that on the clear soup (2.97 ± 1.11 *μ*mol h/L) and raw egg sauce (2.84 ± 1.36 *μ*mol h/L) days, respectively ([Supplementary-material supplementary-material-1]).

**Figure 1:**
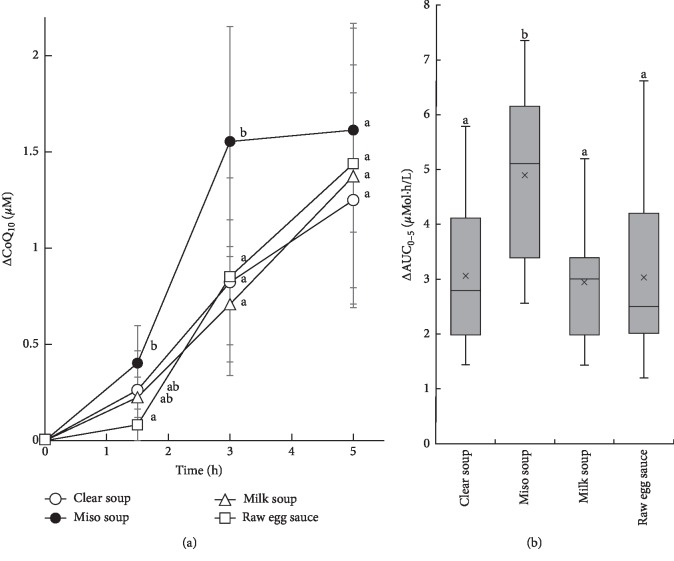
Changes in serum total CoQ_10_ concentration (a) and a comparison of ΔAUC_0–5_ (b) after the consumption of P30 in clear soup, P30 in miso soup, P30 in milk soup, or P30 in raw egg sauce. 120 mg of CoQ_10_ was administered. In Figure 1(a), the open circle represents P30 in clear soup, closed circle represents P30 in miso soup, open triangle represents P30 in milk soup, and open square indicates P30 in raw egg sauce. Data are mean ± SD for the eight individuals who participated in all four of the experiments. Figure 1(b) shows box plots for the ΔAUC_0–5_ for these eight participants. The boundary of the box closest to zero indicates the 25th percentile, the line within the box indicates the median, the multiplication sign within the box indicates the mean, and the boundary of the box farthest from the origin indicates the 75th percentile. Whiskers above and below the box indicate the 10th and 90th percentiles. Data were analyzed with one-way analysis of variance with repeated measures, and differences between the means were evaluated using Holm–Bonferroni post hoc tests. Different lower case letters indicate significant differences, with *P* < 0.05.

Then, we reanalyzed the data from the eight participants (numbers 3, 4, 5, 8, 9, 11, 12, and 13) who had participated in all of the four experiments ([Supplementary-material supplementary-material-1]), using one-way ANOVA with repeated measures (Figure 1). The ΔCoQ_10_ on the miso soup day was significantly higher than on the raw egg sauce days after 1.5 h and higher than on all of the other 3 days 3 h after ingestion (Figure 1(a)). The mean ΔAUC_0–5_ on the miso soup day was 4.94 ± 1.51 *μ*mol h/L, which was 1.6∼1.7-fold higher (*P* < 0.05) than that on the clear soup (3.08 ± 1.33 *μ*mol h/L), milk soup (2.95 ± 1.07 *μ*mol h/L), and raw egg sauce (3.05 ± 1.64 *μ*mol h/L) days, respectively (Figure 1(b)).

These results demonstrate that the absorption rate and bioavailability of supplemental reduced CoQ_10_ up to 5 h after ingestion was increased by suspending it in miso soup. However, this effect was abolished when the CoQ_10_ was suspended in raw egg sauce, even if miso soup was ingested at the same time (experiment 4).

## 4. Discussion

In this study, we determined whether the absorption of a CoQ_10_ supplement would be affected when it was suspended in specific foods. There were no differences in either ΔCoQ_10_ or ΔAUC_0–5_ among participants who consumed a clear soup and test meals on different days, with the exception of the day they consumed the supplement in miso soup. Both the ΔCoQ_10_ at 3 h and the ΔAUC_0–5_ for CoQ_10_ after ingestion of the miso soup was significantly higher than after ingestion of clear soup, milk soup, or raw egg sauce (Figure 1(a)). This result suggests that the suspension of P30 in miso soup may be an effective way of increasing the bioavailability of orally administered CoQ_10_, especially for older people, as the frequency of miso soup consumption is increased with age in Japanese [45].

Interestingly, the effect of miso soup on the absorption of CoQ_10_ was abolished when P30 was suspended in raw egg sauce, despite miso soup also being consumed. This suggests that an interaction between the reduced form of CoQ_10_ and a component of miso might be necessary for the increase in bioavailability of CoQ_10_ achieved by the consumption of miso soup. The absorption of orally administered CoQ_10_ is improved when emulsified with a surfactant that has a higher hydrophile-lipophile balance value [46]. Soy proteins have emulsifying and interfacial properties [47], and the amino-carbonyl reaction of soy proteins with sugar improves their emulsifying properties [48]. Some of the soy proteins in miso are modified by the amino-carbonyl reaction, which explains its brown color. Thus, these modified proteins in miso might be candidates for the components responsible for the enhancement of CoQ_10_ absorption, by emulsification. Besides, miso and soybean extracts improve zinc absorption via increasing cell surface abundance of a zinc transporter [49, 50]. Some components in miso might affect the expression and the activity of a CoQ_10_ transporter protein. However, further studies are required to determine the identity of the active component(s) and the mechanisms involved in the enhanced absorption of CoQ_10_. The miso used in the present study contained only 3.76 and 0.02 *μ*g of the reduced and oxidized forms of CoQ_10_ per g miso (data not shown), and 10 g of the miso was used for the preparation of one serving of miso soup; therefore, it is unlikely that the CoQ_10_ contributed by the miso is involved in the enhancement in bioavailability of CoQ_10_.

We also estimated the ΔAUC value for the reduced form of CoQ_10_ in the previously published study to compare these with the ΔAUC_0–5_ values obtained in the present study. Hosoe et al. performed a single-dose experiment after meal ingestion using 150 mg of the reduced form of CoQ_10_ in a soft capsule [27]. The estimated ΔAUC for the first 6 h after ingestion (ΔAUC_0–6_) in this study was 2.9 *μ*mol h/L, whereas the ΔAUC_0–5_ in our control experiment, in which 120 mg of the reduced form of CoQ_10_ was consumed in a clear soup was 2.97 ± 1.11 *μ*mol h/L (Experiment 1 in [Supplementary-material supplementary-material-1]), suggesting that similar results can be obtained when the reduced form of CoQ_10_ is administered. The ΔAUC_0–5_ associated with P30-plus-miso ingestion (4.44 ± 1.40 *μ*mol h/L, Experiment 2 in [Supplementary-material supplementary-material-1]) was >1.5 times higher than those associated with the ingestion of other foods (clear soup, milk soup, and raw egg sauce), implying that the ingestion of CoQ_10_ suspended in miso soup is one of the best ways of increasing its bioavailability, at least, when P30 is used.

One limitation of our study was that the serum concentrations of CoQ_10_ were determined only up to 5 h after ingestion because of the research environment and availability of the participants. In contrast, in most previous studies, these concentrations were determined up to 12 or 24 h after a single dose of CoQ_10_. One could argue that miso soup may accelerate rather than enhance CoQ_10_ absorption, as the maximum CoQ_10_ concentration or the maximum CoQ_10_ concentration-time could not be determined. The half-life of CoQ_10_ in plasma may reach 33 h, and 5 to 6 days were required for plasma CoQ_10_ levels to return to baseline following a single dose [51]. The possibility of overestimation of the effect of miso soup on CoQ_10_ absorption cannot be denied.

Another limitation of our study was that there is no negative control, i.e., meals alone. In the previous reports, there was no placebo control for the determination of the absorption/bioavailability of CoQ_10_ supplements [25–27, 52–55]. Also, the effect of CoQ_10_ contents in the provided meals seemed to be extremely low as the estimated CoQ_10_ amount was less than one two-hundredth of the CoQ_10_ supplements ingested. However, these remain in a matter of speculation.

To further characterize the effects of miso soup ingestion on the bioavailability of CoQ_10_, it would be of interest to compare the serum concentrations of CoQ_10_ after multiple meals of miso soup or water containing P30 with or after meals for several weeks. The significance of the present findings will be evaluated by performing such a clinical experiment in the near future.

## 5. Conclusions

In conclusion, the ingestion of a granulated and reduced form of CoQ_10_ in miso soup increased the bioavailability of CoQ_10_ by ∼1.5 times over its ingestion under the other conditions tested. Thus, the absorption of a CoQ_10_ supplement can be enhanced by combining it with specific food substances, such as miso, in addition to taking it with a meal *per se*.

## Figures and Tables

**Table 1:** Meal menus for each experiment.

Experiment no.	1	2	3	4
Experiment	Clear soup	Miso soup	Milk soup	Raw egg sauce

*Meal menu*				
Main dish	Grilled salmon with Japanese radish marinated in citrus juice			
Side dish	Boiled spinach flavored with bonito flakes and soy sauce			
Staple food	Rice	Rice	Rice	Rice with stirred raw egg sauce^a^
Soup	Clear soup^a^	Miso soup^a^	Milk soup^a^	Miso soup
Other	—	—	—	—

*Nutrient content per serve*				
Energy (kcal)	336	356	437	430
Protein (g)	20.1	21.5	25.1	27.5
Fat (g)	3.6	4.3	9.3	9.4
Carbohydrate (g)	54.1	56.5	61.3	56.4
CoQ_10_ (mg)^b^	120	120	120	120

^a^P30 was suspended in this food item; ^b^only CoQ_10_ obtained from P30 is recorded.

## Data Availability

The data used to support the findings of this study are included within the article and also available upon request.

## Supplementary Materials

Supplementary Materials Figure S1: Meals provided to the participants. Figure S2: Changes in serum total CoQ_10_ using unpaired one-way ANOVA. Table S1: Participation in each of the four experiments. Table S2: The increase in serum total CoQ_10_ concentration of each subject after the consumption of each food type.
