# Supplementary material for: Miso Soup Consumption Enhances the Bioavailability of the Reduced Form of Supplemental Coenzyme Q10
Source: J Nutr Metab. 2020 Jan 7;2020:5349086. doi: 10.1155/2020/5349086 (PMC6969983; doi:10.1155/2020/5349086)
Supplement: Supplementary Materials — Figure S1: Meals provided to the participants. Figure S2: Changes in serum total CoQ10 using unpaired one-way ANOVA. Table S1: Participation in each of the four experiments. Table S2: The increase in serum total CoQ10 concentration of each subject after the consumption of each food type. [file 5349086.f1.pdf]

Figure. S1

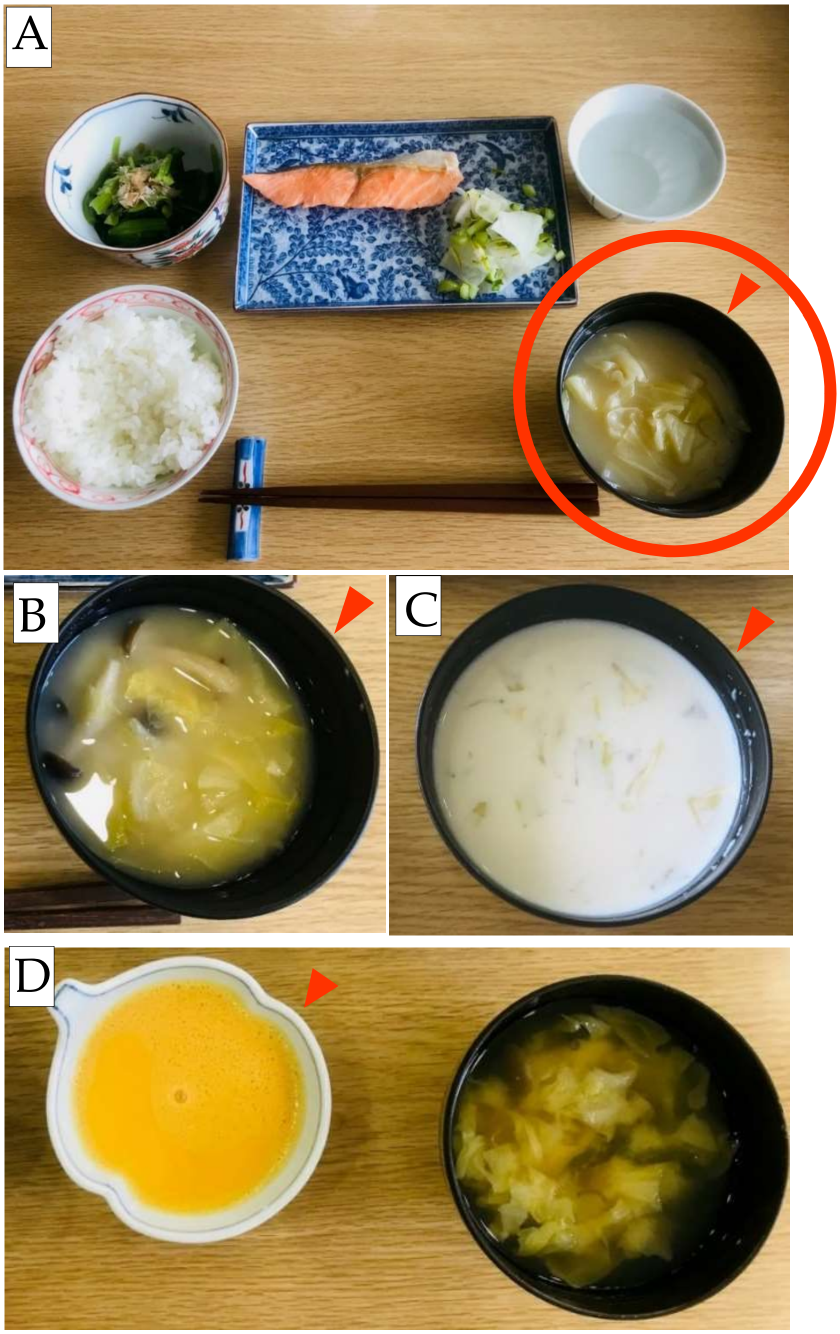

Figure S1. Meals provided to the participants.
A, A basic meal, consisting of grilled salmon with Japanese radish marinated in citrus juice (upper right), boiled spinach flavored with bonito flakes and soy sauce (upper left), rice (lower left), and P30-supplemented clear soup with cabbage and mushrooms tasted with salt (lower right, framed by a red circle). The clear soup was replaced with P30-supplemented dishes (B-D) when the test experiments were performed. B, miso soup. C, milk soup. D, stirred raw egg sauce and miso soup. Red arrowheads indicate P30-supplemented menus, which contain 120 mg per serve of the reduced form of CoQ_{10}.

Figure. S2

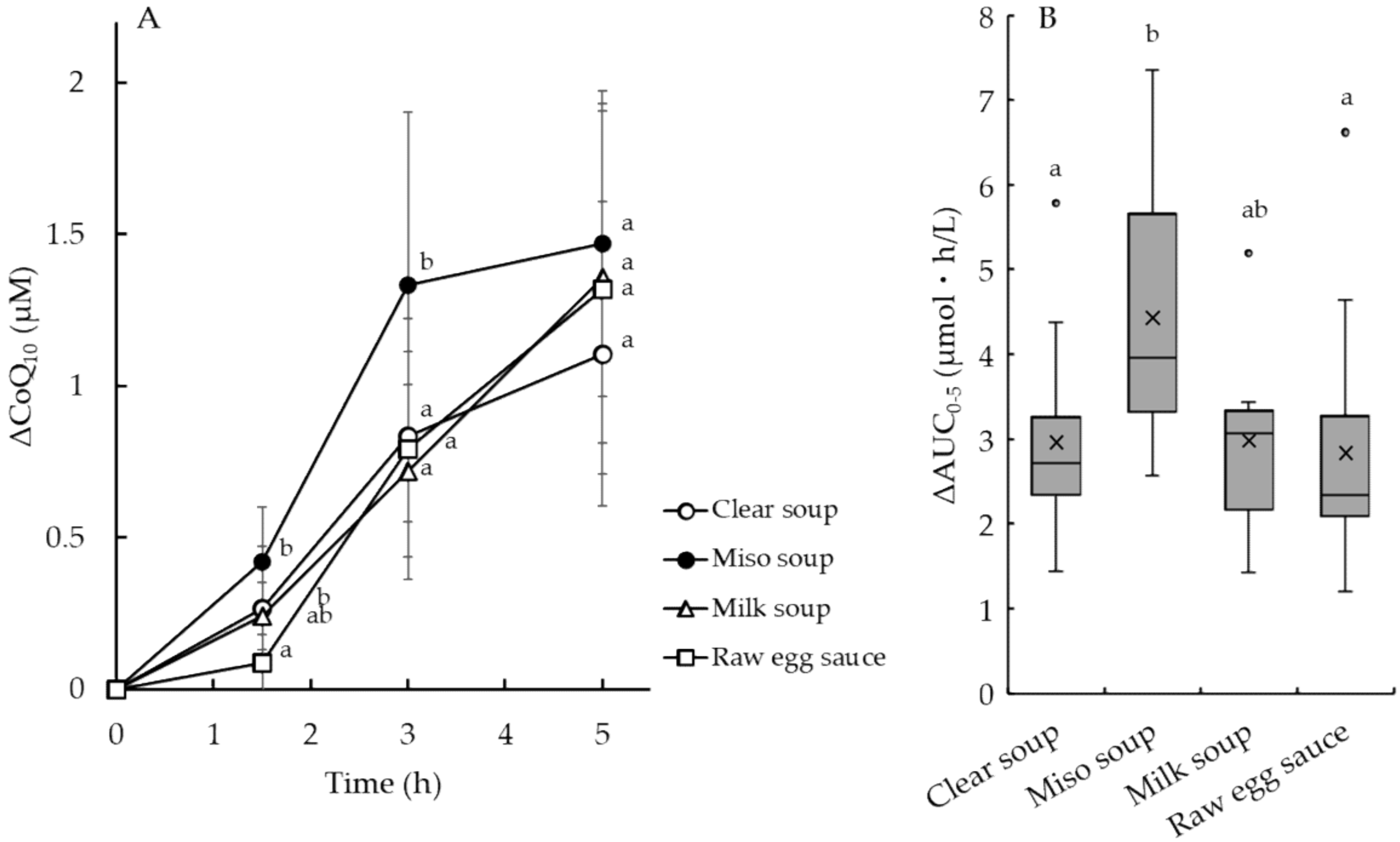

Figure S2. **Changes in serum total CoQ_{10} concentration (A) and comparison of $\Delta AUC_{0\text{-}5}$ (B) using unpaired one-way ANOVA.**
A, open circle, P30 in clear soup (n=12); closed circle, P30 in miso soup (n=13); open triangle, P30 in milk soup (n=9); open square, P30 in raw egg sauce (n=13). Data are mean ± SD. B, Box plots of the areas under the serum CoQ_{10} concentration-time curves for the 5 h after ingestion ($\Delta AUC_{0\text{-}5}$) from all subjects. The boundary of the box closest to zero indicates the 25th percentile, the line within the box indicates the median, the multiplication sign within the box indicates the mean, and the boundary of the box farthest from the origin indicates the 75th percentile. Whiskers above and below the box indicate the 10th and 90th percentiles, and the points above and below the whiskers indicate outliers (outwith the 10th and 90th percentiles). Because the number of participants differed in each experiment, the data were analyzed using unpaired one-way ANOVA and the differences between the means were evaluated using Holm-Bonferroni *post hoc* tests. Different lowercase letters indicate significant differences, $P<0.05$.

Table S1. Participation in each of the four experiments.

Experiment No.			1	2	3	4	Data used in unpaired one-way ANOVA	Data used in one-way ANOVA with repeated measures
Participant			Clear soup	Miso soup	Milk soup	Raw egg sauce		
ID	Sex	Age						
1	Female	21	○	○		○	○	
2	Female	21	○	○		○	○	
3	Female	21	○	○	○	○	○	○
4	Female	21	○	○	○	○	○	○
5	Female	21	○	○	○	○	○	○
6	Female	21	○	○		○	○	
7	Female	22		○	○	○	○	
8	Female	22	○	○	○	○	○	○
9	Female	22	○	○	○	○	○	○
10	Female	24	○	○		○	○	
11	Female	32	○	○	○	○	○	○
12	Female	28	○	○	○	○	○	○
13	Male	52	○	○	○	○	○	○
Number of Participants			12	13	9	13	13	8

Table S2. The increase in serum total CoQ_{10} (ΔCoQ_{10}) concentration and $\Delta AUC_{0–5}$ of each subject after the consumption of each food type

(Experiment 1. Clear soup)

ID	CoQ_{10} at 0 h (μmol/L)	ΔCoQ_{10} (μmol/L)				ΔAUC_{0-5} (μmol.h/L)
		0 h	1.5 h	3 h	5 h	
1	0.79	0.00	0.09	0.87	0.81	2.46
2	0.75	0.00	0.62	0.83	0.72	3.10
3	0.45	0.00	0.14	0.55	0.27	1.44
4	0.70	0.00	0.19	0.86	1.53	3.31
5	0.93	0.00	0.50	1.14	1.62	4.37
6	0.57	0.00	0.17	1.09	0.81	2.98
7						
8	0.62	0.00	0.12	0.67	0.98	2.34
9	0.77	0.00	0.68	1.48	2.18	5.78
10	0.61	0.00	0.23	0.63	0.93	2.38
11	0.53	0.00	0.21	0.40	0.86	1.86
12	0.47	0.00	0.25	0.64	0.95	2.45
13	0.95	0.00	0.01	0.85	1.61	3.13
Mean±SD	0.68±0.16	0	0.27±0.20	0.84±0.28	1.11±0.50	2.97±1.11

(Experiment 2. Miso soup)

ID	CoQ_{10} at 0 h (μmol/L)	ΔCoQ_{10} (μmol/L)				ΔAUC_{0-5} (μmol.h/L)
		0 h	1.5 h	3 h	5 h	
1	0.80	0.00	0.36	0.83	1.64	3.63
2	0.79	0.00	0.45	0.64	1.07	2.89
3	0.51	0.00	0.55	1.68	1.57	5.33
4	0.73	0.00	0.27	1.12	1.60	3.97
5	1.12	0.00	0.43	2.21	1.71	6.21
6	0.83	0.00	0.21	1.29	0.89	3.47
7	0.86	0.00	0.56	1.26	1.69	4.73
8	0.99	0.00	0.56	2.29	1.13	5.98
9	1.00	0.00	0.37	2.21	2.92	7.35
10	0.73	0.00	0.65	0.89	0.91	3.45
11	0.70	0.00	0.37	0.87	1.14	3.21
12	0.67	0.00	0.68	1.38	1.47	4.89
13	1.05	0.00	0.00	0.69	1.37	2.57
Mean±SD	0.83±0.17	0	0.42±0.18	1.33±0.57	1.47±0.50	4.44±1.40

(Experiment 3. Milk soup)

ID	CoQ_{10} at 0 h (μmol/L)	ΔCoQ_{10} (μmol/L)				ΔAUC_{0-5} (μmol.h/L)
		0 h	1.5 h	3 h	5 h	
1						
2						
3	0.56	0.00	0.30	0.80	1.08	2.93
4	0.60	0.00	0.18	0.85	1.67	3.44
5	1.20	0.00	0.24	1.24	2.66	5.20
6						
7	0.64	0.00	0.38	0.82	1.23	3.23
8	0.55	0.00	0.11	0.28	0.76	1.43
9	0.88	0.00	0.31	0.63	1.66	3.23
10						
11	0.49	0.00	0.33	0.26	0.83	1.78
12	0.50	0.00	0.31	0.86	1.10	3.07
13	0.72	0.00	0.02	0.73	1.24	2.55
Mean±SD	0.68±0.22	0	0.24±0.11	0.72±0.29	1.36±0.55	2.98±1.02

(Experiment 4. Raw Egg Sauce)

ID	CoQ_{10} at 0 h (μmol/L)	ΔCoQ_{10} (μmol/L)				ΔAUC_{0-5} (μmol.h/L)
		0 h	1.5 h	3 h	5 h	
1	1.07	0.00	0.00	0.73	0.79	2.06
2	0.87	0.00	0.22	1.10	1.40	3.66
3	0.64	0.00	0.01	0.85	1.36	2.87
4	0.65	0.00	0.20	0.45	0.89	1.98
5	0.93	0.00	0.21	1.85	3.06	6.62
6	0.94	0.00	0.23	0.56	0.90	2.21
7	0.92	0.00	0.00	0.54	1.30	2.24
8	0.70	0.00	0.10	0.72	1.21	2.62
9	1.11	0.00	0.00	1.52	1.98	4.64
10	0.75	0.00	0.05	0.57	1.27	2.34
11	0.76	0.00	0.11	0.32	0.48	1.20
12	0.82	0.00	0.02	0.61	1.27	2.37
13	1.01	0.00	0.00	0.49	1.26	2.12
Mean±SD	0.86±0.15	0	0.09±0.09	0.79±0.43	1.32±0.61	2.84±1.36
